# Identifying the data elements and functionalities of clinical decision support systems to administer medication for neonates and pediatrics: a systematic literature review

**DOI:** 10.1186/s12911-023-02355-5

**Published:** 2023-11-16

**Authors:** Somaye Norouzi, Zahra Galavi, Leila Ahmadian

**Affiliations:** Department of Health Information Sciences, Faculty of Management and Medical Information Sciences, Kerman University of Medical Sciences, Kerman, Iran

**Keywords:** Clinical decision support system, Medication administration, Data element, HL7 FIHR, Functionality, Systematic literature review

## Abstract

**Background:**

Patient safety is a central healthcare policy worldwide. Adverse drug events (ADE) are among the main threats to patient safety. Children are at a higher risk of ADE in each stage of medication management process. ADE rate is high in the administration stage, as the final stage of preventing medication errors in pediatrics and neonates. The most effective way to reduce ADE rate is using medication administration clinical decision support systems (MACDSSs). The present study reviewed the literature on MACDSS for neonates and pediatrics. It identified and classified the data elements that mapped onto the Fast Healthcare Interoperability Resources (FHIR) standard and the functionalities of these systems to guide future research.

**Methods:**

PubMed/ MEDLINE, Embase, CINAHL, and ProQuest databases were searched from 1995 to June 31, 2021. Studies that addressed developing or applying medication administration software for neonates and pediatrics were included. Two authors reviewed the titles, abstracts, and full texts. The quality of eligible studies was assessed based on the level of evidence. The extracted data elements were mapped onto the FHIR standard.

**Results:**

In the initial search, 4,856 papers were identified. After removing duplicates, 3,761 titles, and abstracts were screened. Finally, 56 full-text papers remained for evaluation. The full-text review of papers led to the retention of 10 papers which met the eligibility criteria. In addition, two papers from the reference lists were included. A total number of 12 papers were included for analysis. Six papers were categorized as high-level evidence. Only three papers evaluated their systems in a real environment. A variety of data elements and functionalities could be observed. Overall, 84 unique data elements were extracted from the included papers. The analysis of reported functionalities showed that 18 functionalities were implemented in these systems.

**Conclusion:**

Identifying the data elements and functionalities as a roadmap by developers can significantly improve MACDSS performance. Though many CDSSs have been developed for different medication processes in neonates and pediatrics, few have actually evaluated MACDSSs in reality. Therefore, further research is needed on the application and evaluation of MACDSSs in the real environment.

**Protocol Registration:**

(dx.doi.org/10.17504/protocols.io.bwbwpape).

**Supplementary Information:**

The online version contains supplementary material available at 10.1186/s12911-023-02355-5.

## Introduction

Patient safety is a central healthcare policy worldwide. It is defined by the World Health Organization (WHO) as the prevention of errors and adverse effects for patients who receive care services [1]. As the American Medical Association (AMA) reported, human errors account for most threats to patient safety [2]. Adverse drug events (ADE) are among the main threats to patient safety during hospitalization and can lead to delayed discharge (from the hospital) and higher service costs [3, 4]. Unintended events only happen rarely, but the drug prescription and administration process are highly risky. ADE can threaten children more due to their physiological condition and body growth [5].

The results of a systematic review showed that ADE rate was three-fold in pediatrics compared to adults [6]. A body of research showed that, in medication administration, children are considered a vulnerable group of patients [7]. Children are at a higher risk of ADE in each stage of the medication management process because prescribing, dispensing, and administering drugs for children require better estimation than for adults [5]. A systematic review in 2013 showed that about 26.9% of hospital errors occurred during medication administration in pediatrics [7]. As American MEDMARX reported, 21%, 22%, and 33% of adverse events occurred in prescription, medication delivery, and medication administration [8]. Administration is the final stage of a medication process in which nurses and patients are directly involved. It is also the last stage of protection to prevent potential unintentional consequences for patients [9].

There are several ways to reduce the rate of ADE. Among the most effective is using a clinical decision support system (CDSS) [10]. Overall, many CDSSs have been developed to reduce ADE rates in pediatrics and neonates. They have proved effective in the prescription stage. The primary users of CDSSs are physicians [11–13]. The ADE rate is high in the administration stage, as the final stage of preventing medication error in pediatrics and neonates [7, 9]. Thus, implementing a CDSS for nurses can be effective in administering proper medications for pediatrics and neonates [14].

The development of medication administration clinical decision support systems (MACDSS) for pediatrics and neonates is quite a challenge. Any failure can put the patient’s life or the health system at risk [15]. It is essential to identify the useful data elements and functionalities to have an effective and well-developed CDSS. To implement a new MACDSS, it is necessary to identify the related literature and determine the data elements and functionalities needed to develop these systems. To have a set of data elements with the same format, they can be mapped onto standards. Using standards such as FHIR at the outset of system development can accompany the syntactic interoperability of systems. FIHR standard determines data elements in the healthcare domain to facilitate data sharing and integrating health information systems [16].

To our best knowledge, no systematic review has been conducted to identify the data elements mapping onto the FHIR standard and functionalities of MACDSS for neonates and pediatrics. The existing systematic reviews have only addressed interventions to reduce the rate of medication errors in children [7, 17]. In their systematic review and meta-analysis, Berdot et al. investigated interventions to reduce the rate of nurses’ medication administration errors in different inpatient conditions [18]. Similarly, Moore et al. published a systematic review of the effect of health information technology (HIT) on nurses’ timing in different inpatient settings [19]. Gates et al. conducted a systematic review and meta-analysis to compare the prevalence and impact of the adverse events of dose errors in pediatric settings with/without HIT [20].

Another systematic review analyzed the process of CDSSs development, functionalities, and features in patients with chronic diseases [15]. Two more systematic reviews analyzed the effect of HIT functionalities on patient outcomes [21, 22]. Tummers et al. mentioned not considering operational functionality as a barrier to inappropriate implementation of health information systems [23].

A health information system supporting decision-making can significantly improve nurses’ performance and can facilitate medication administration process [14]. The present study reviewed the literature to identify the data elements mapped onto the FHIR standard. It also aimed to identify functionalities used to design MACDSS for pediatrics and neonates [24]. Identifying data elements and functionalities of MACDSS can help health developers and policymakers in the design process [25]. The present research can help design an optimal MACDSS.

## Materials and methods

### Protocol registration and amendment

We conducted a systematic literature review (SLR) in accordance with the Preferred Reporting Items for Systematic Reviews and Meta-analyses (PRISMA) guidelines [26] (see S File 1). Our protocol was registered in Protocols.io (dx.doi.org/10.17504/protocols.io.bwbwpape) [27].

### Inclusion and exclusion criteria (eligibility criteria)

The present SLR included primary studies that had designed MACDSS for pediatrics and neonates (population below age 18) regardless of the design platform. Those addressing system design for a specific drug category in pediatrics and neonates were included. All papers described system design for medication administration in all pediatric and neonate settings, including general pediatric, NICU, PICU, pediatric emergency, and pediatric oncology. The papers included met the conditions mentioned above and had nurses as their primary users.

The related theses or conference papers that met the eligibility criteria were included. Those regarding the barcode medication administration systems and the pediatric nutrition informatics domain were excluded. Letters to editorials, protocol studies, commentaries, studies with no full text, and opinion articles were excluded. The primary papers published between 1995 and 2021 were included with no limitation set on language.

### Search methods and resources (search strategy)

Four databases were searched including PubMed/ MEDLINE, Embase via Embase.com, and CINAHL (Cumulative Index to Nursing and Allied Health Literature) via EBSCOhost. ProQuest was an additional source to capture any relevant thesis. Papers published between January 1, 1995, and June 31, 2021, were retrieved. The latest search was run on July 8, 2021. Also, in addition, we scanned the reference lists of all included papers to find relevant papers.

To set the relevant key terms, Medical Subject Headings (MeSH), Embase subject headings (EMTREE), and the keywords mentioned in other related papers were reviewed. A panel of experts read and commented on the extracted key terms. Two categories of key terms were defined, the terms related to health information technology (HIT) and those related to medication administration. For the search, the operator OR was used for each category. Then, the terms within the two categories were searched together with the operator AND (see S File 2).

### Study selection

All primary papers were included regardless of the design. The duplicates were excluded, and the remaining papers were imported to the Rayyan website [28] for a screen-check. Based on the eligibility criteria, they were checked independently by two subject experts (SN and ZG). After title and abstract screening, the full texts were independently reviewed by the same reviewers to select the relevant papers. Disagreements were solved through discussions. A third reviewer (LA) was consulted if no consensus was made. The reasons for exclusion were documented.

### Data extraction

In this step, a customized data extraction sheet was created and completed by two reviewers (SN, ZG) independently. The following data were collected from the included papers: first author’s name, year of publication, country, objectives, type of study, setting, design platform, key findings, and drug category. The design platform of information systems in the included papers was categorized into three types, *mobile application, computer application,* and *web application*.

The data elements and functionalities were underrepresented in the full texts of the papers. Also, figures, charts, and tables were used to identify the data elements and functionalities. In the end, the extracted data elements were mapped onto the FHIR standard [16]. The extracted functionalities were further divided into *general* and *specific MACDSS*. The former referred to functionalities used in other CDSSs. In this step, any disagreement was resolved through reviewers’ discussions. When needed, a third reviewer was consulted.

### Synthesis of results

After the initial data extraction, thematic analysis was used for data synthesis. Initially, data elements and functionalities were extracted based on words, descriptions, and concepts expressed in the full texts of the included studies (figures, charts, and tables). After carefully analyzing the data elements, it was decided to map them onto the FIHR standard to ensure consistency. Through identifying the similarities and differences between data elements, a set of data elements was created to adhere to the standard and easily integrate into other systems. This process was crucial to ensure accuracy and efficiency of the data management system. The extracted data (data elements and functionalities) were categorized using thematic analysis. As this study was SLR, no meta-analysis could be conducted because of the methodological heterogeneity of papers.

### Assessment of the level of evidence

The present study was SLR, and the included papers were heterogeneous in terms of search methodology; Thus, checklists such as Cochrane, JBI, and CASP could not be used [29–31]. Thus, a similar approach to what Poissant et al. and Moore et al. used in their research to assess the quality of papers was employed [19, 32]. At first, the methodologies used in papers were analyzed by two reviewers (SN and ZG). Next, the papers were categorized according to the level of evidence ([33]; www.cebm.ox.ac.uk/resourcees/levels-of-evidence/oxford-centre-for-evidence-based-medicine-levels-of-evidence-march-2009). A third expert reviewer (LA) was consulted in case of any disagreement.

**Fig. 1:**
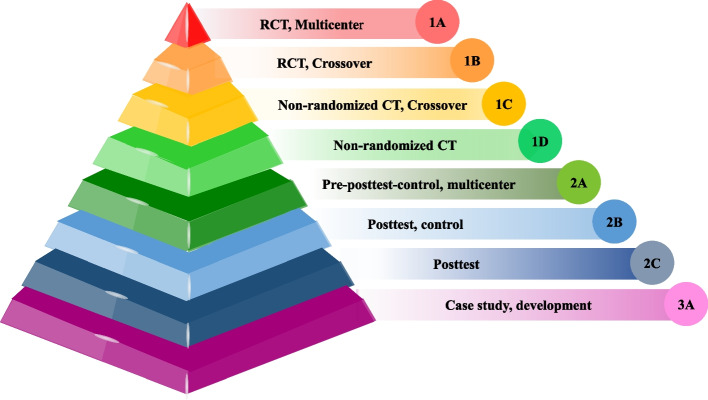
Level of Evidence

The first category is *clinical trials (CT)* in the evidence pyramid (Fig. 1). The first level of this category comprises *Multicenter randomized control trials (RCT)* at the top of pyramid. According to the study design (randomization or cross-over), the remaining CT lie at lower levels. Another category is observational studies, divided into three levels. The highest level includes studies with a *multicenter pretest and posttest design with a control group*. The next level includes studies with a posttest and a control group. The next one includes studies with only a posttest. The last level consists of case studies.

Besides analyzing the methodologies of papers, we assessed them in terms of the data collection procedure. The data collection methods were of four types: *“medication error calculation in the real environment,” “medication error calculation in the simulated environment,” “time and motion observed/video recording,” and “evaluation by self-report/survey.”*

## Results

**Fig. 2:**
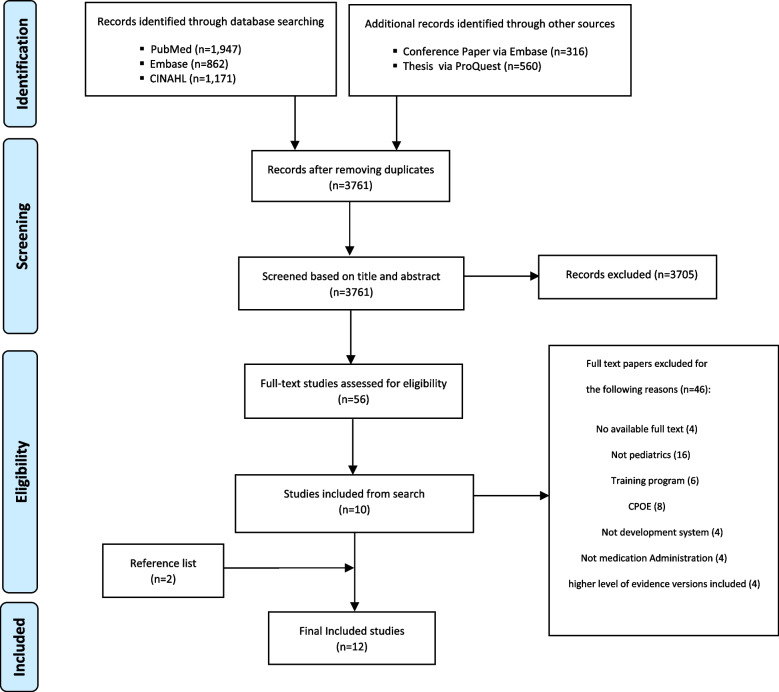
PRISMA flow diagram

In the initial search, 4,856 papers were identified. After removing duplicates, 3,761 titles and abstracts remained according to the eligibility criteria. Finally, 56 full-text primary studies were retained for evaluation. The full-text review of papers led to the retention of 10 papers which met the inclusion criteria. In addition, two papers from the reference lists were included. In total, 12 studies were analyzed in this review [34–45]. Although some met the inclusion criteria [46–49], they were eventually left out because their higher level of evidence versions were already included. Some data elements and functionalities were extracted from these papers in the data extraction stage (Fig. 2).

### Paper descriptions

The general characteristics of papers are outlined in Table 1. The most important features are the purpose of study, detailed technology, target population, setting, and name of the guideline/standard used in the system.

**Table 1:** Characteristics of the papers included (*n* = 12)

**Study**	**Type of study**	**Objective (s)**	**Country**	**Type of institution**	**Integrated/** **standalone system**	**System developer**	**User**	**Study setting**	**Type of medications**	**Name of guideline/** **standard used in the system**	**Key finding(s)**	**Level of Evidence**
**Siebert et al. (2019) **[37]	RCT, Multi center	To assess a mobile device application during simulation-based resuscitations in various hospital settings	Switzerland	Academic	Standalone	Homegrown	Nurse	Pediatric emergency	Resuscitation drugs,15 drugs for continuous infusion, 19 drugs for direct intravenous injection	American Heart Association pediatric cardiac arrest algorithm	Medication errors, mean time to drug preparation and drug delivery were reduced	1A
**Parush et al. (2020) **[45]	Non- randomized CT, Cross over	to design and empirically test a graphic dosage calculator tailored for pediatric medication calculation in prehospital emergency care	Israel	Academic	Standalone	Homegrown	Paramedics	Pediatric emergency	pediatric emergency drugs	N/A	significantly decreasing time to calculate doses with the graphic calculator compared with the handbook	1C
**Ni et al. (2018) **[42]	Posttest	Specific aims: 1. to develop an automated system utilizing comprehensive EHR information to detect dosing related medication error in real time, 2. To prospectively evaluate system performance in NICU prior to clinical integration, and 3. to estimate the system’s potential to mitigate MAE harm for neonatal patients	USA	Academic	Integrated	Homegrown	Clinician	NICU	10 high-risk continuous intravenous infusions and medications. TPN, lipids, intravenous fluids, insulin, morphine, fentanyl, milrinone, vasopressin, dopamine, and epinephrine	NCC MERP^a^	improving significantly MAE detection by the system. decreasing the time of patient exposure to harm due to drug errors by the system	2C
**Zahn et al. (2021) **[39]	Case study, development	to describe the development and history of the pediatric drug information system (PDIS) for Germany and its evaluation by German healthcare professionals	Germany	Academic	Standalone	Homegrown	Clinician	General pediatric	N/A	SmPC (Summary of Product Characteristics.), ATC-code	N/A	3A
**Reynolds et al. (2019) **[41]	Multicenter pretest–posttest, control	to evaluate end-user acceptance and the effect of a commercial handheld decision support device in pediatric intensive care settings	USA	Non-academic	Standalone	Commercial	Nurse	NICU, PICU	intravenous and other liquid medications	NCC MERP^a^	this study did not reveal significant differences in cognitive load and administration errors after deploying the system	2A
**Dodson et al. (2021) **[36]	Case study, development	to evaluate the perceptions of a prototype of a clinical decision support tool through a mobile application for pharmacokinetics	USA	Academic	Standalone	Homegrown	Nurse	General pediatric	N/A	N/A	N/A	3A
**Ateya et al. (2017) **[44]	Posttest, control	To describe the insulin calculator tool, workflow, and satisfaction of clinical users and their perception of its impact on work efficiency, and quality of patient care, and measure its impact on the incidence of hypoglycemia to assess the safety of its utilization	USA	Academic	Integrated	Homegrown	Nurse	General pediatric	Insulin	N/A	there was no significant difference in hypoglycemia rates, severe hypoglycemia rates and length of stay by using the system.	2B
**Levy et al. (2011) **[40]	Case study, development	to describe the experience in relation to the deployment of the system, including integration of multiple clinical information Systems and oncology-specific configurations	USA	Academic	Integrated	Homegrown	Nurse	Pediatric chemotherapy	N/A	ASCO/ONS guidelines for Chemotherapy administration	N/A	3A
**Bury et al. (2005) **[38]	RCT, cross over	to describe the design, implementation, and preliminary evaluation of the LISA system	UK	Academic	Integrated	Homegrown	Clinician	Pediatric chemotherapy	Oral chemotherapy	PROforma	using LISA reduced the time novices, while increasing the time taken by experts and did not have a significant impact on the time taken by intermediates in dose adjustment decreasing error in dose calculation	1B
^a^**Damhoff et al. (2014) **[43]	Non-randomized CT, Crossover	to assess the accuracy of the eBroselow system and the time needed to prepare medications during pediatric simulated resuscitations compared with standard dosing references	USA	Academic	Standalone	Commercial	Nurse	Pediatric emergency	Pediatric emergency drugs	NDC (National drug code)	Elimination of dose calculation errors by the system, decreasing the time to prepare medications	1C
**Shannon et al. (2002) **[35]	Non-randomized CT	to design a computerized system for calculating resuscitation requirements and testing this system to ensure that it gives accurate and fast results	UK	Non- academic	Standalone	Homegrown	Clinician	Pediatric emergency	10 different drugs for resuscitation and Antibiotic	N/A	decreasing dose errors by the system, decreasing the time to prepare medications	1D
**Ellis et al** **(2012) **[34]	Non-randomized CT, Crossover	to assess whether a graphic dose calculator, in comparison to standard paper/pencil and calculator, can support the double-checking process and reduce the rate of potential errors with high-alert drugs	Canada	Academic	Standalone	Homegrown	nurse, student nurse	Pediatric emergency	Intravenous morphine	Lexicomop /Micromedex (2011)	no significant difference to detecting error by the system and traditional method No difference to take the time to preparing medication in two groups	1C

^a^Some characteristics were extracted from the App. National Coordinating Council for Medication Error Reporting and Prevention Index. Medication administration error

### Technology characteristics

Most studies (*n* = 10) developed their system as homegrown in academic hospitals. Two (*n* = 2) used commercial systems in a non-academic environment. Eight (*n* = 8) developed standalone systems, and four (*n* = 4) were integrated with hospital information systems. Among all, three (*n* = 3) implemented their system as a mobile application. The other four (*n* = 4) implemented their systems on the web. In three studies (*n* = 3), the system design platforms were computer applications. They also designed a system with different platforms integrated into the existing hospital systems [42] (Tables 1 and 3). Among all included studies, three [34, 39, 43] used the ATC-code, Micromedex, and national drug code (NDC) terminologies [50].

### Target population & setting characteristics

The papers included investigations of different pediatric and neonatal settings. Five were conducted in the pediatric emergency setting and two in the pediatric chemotherapy setting. One was conducted simultaneously in the NICU and PICU settings. Others were carried out in other pediatric and neonatal settings. More than half of system users were nurses in different pediatric and neonatal settings (*n* = 7). Other users were clinicians (*n* = 4), and paramedics (*n* = 1). Six studies (*n* = 6) were conducted in the USA, two (*n* = 2) in the UK, four (*n *= 4) in Canada, Israel, Switzerland, and Germany.

### Data elements extracted from the included studies mapped onto the FHIR standard

**Fig. 3:**
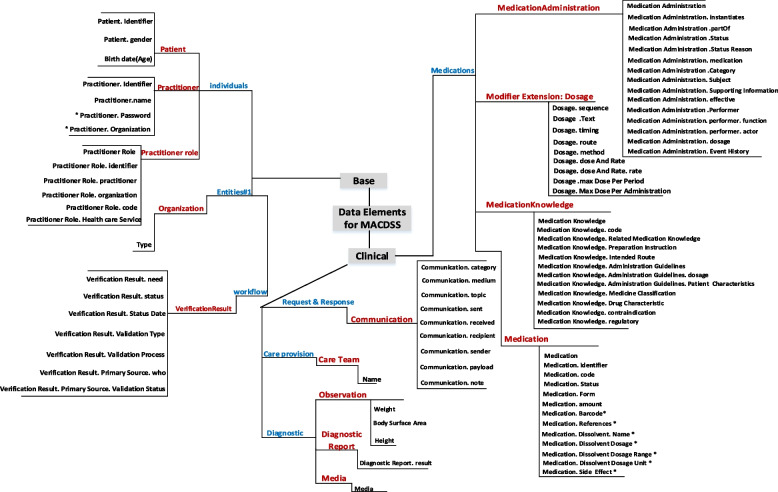
Classification map, an overview of the identified data elements for the MACDSS mapped onto the FHIR standard. * The data elements are related to the researcher’s extension

Figure 3 and S Table 1 show a complete list of data elements extracted from the studies. The extracted data elements were divided into *base and clinical* categories in the FHIR standard. Seven items were selected from the subcategories. Next, 14 resources of these subcategories were used. A total number of 84 data elements were extracted and mapped into these resources. Nine data elements were extracted from the profile extensions of this standard. If the data element was not mapped to any of the resources within this standard, it was placed as an extension in the most relevant resource. Nine data elements were identified this way.

### Functionalities extracted from the included studies

Table 2 and S File 3 show a complete list of extracted functionalities and their definitions. In total, 18 functionalities were extracted from the papers. Eleven functionalities were categorized as specific for MACDSS and seven others as general CDSS.

**Table 2:** List of functionalities extracted from papers (*n* = 12) (see S File 3 for Functionalities definitions)

**Functionality**	**Setting**					**Type of functionality**	
	**Pediatric Emergency**	**NICU**	**PICU**	**Pediatric Chemotherapy**	**General Pediatric**	**General**	**Specific to MACDSS**
**Drug dosage calculation**	√ [34, 35, 37, 43, 45]	√ [41]	√ [41]	√ [38]	√ [36, 44]		√
**Calculating dilution of drug and Dose volume**	√ [34, 35, 37, 43, 45]	√ [41]	√ [41]				√
**Calculating drug rates of infusion**	√ [37, 43, 45]	√ [41]	√ [41]				√
**Keeping the history of information in system**	√ [37]	√ [41, 42]	√ [41]	√ [38, 40]		√	
**Using alarms and alerts in the** **system & type of alert**	√ [37] Alarm for validating selection of look-alike/ sound-alike medication	√ [42] Real-time notification about medication error event	√ [41] Alert for out of range dose-alert for high alerts medications	√ [38] Weekly advice on modifications of oral chemotherapeutic agents, and rational for its proposal	√ [44] Alert for out of range insulin dose	√	
	√ [34] Alert for out-of-range dose	√ [41] Alert for out of range dose -Alert for high alerts medications		√ [40] Alert for missing order, Wrong Dose, Wrong route, Wrong schedule			
**Displaying the status of stages of the medication use process**	√ [37]	√ [41]	√ [41]	√ [40]			√
**Having the capability to share** **information with other institutions**	√ [37, 43]					√	
**Having the capability to edit drug information in the system**	√ [37]	√ [41]	√ [41]	√ [38, 40]		√	
**Providing a visual image and color** **text in the system**	√ [34, 37, 45]	√ [41, 42]	√ [41]		√ [36]		√
**Providing additional information** **about medicine to the user**	√ [34, 43]	√ [41]	√ [41]		√ [36, 39, 44]		√
**Having the capability to clear information in the system**	√ [34, 43, 45]					√	
**Having the capability to search information in the system**	√ [43, 45]	√ [41]	√ [41]			√	
**Having the capability to log in/out of the system**	√ [43]			√ [38]		√	
**Having the capability to lookup by barcode in the system**	√ [37, 43, 44]						√
**Having the capability to modify dose in the system**	√ [37]						√
**Displaying drug administration route**	√ [35, 37, 43, 45]	√ [41]	√ [41]	√ [38]			√
**Displaying dose form/dosage regimen**	√ [37, 45]	√ [41]	√ [41]		√ [36, 39, 44]		√
**Displaying medication preparation steps**	√ [37, 45]	√ [41]	√ [41]				√

In most papers (*n* = 9), "*drug dosage calculation"* functionality was identified. All studies in the pediatric emergency setting included this functionality in their system. Six studies (*n* = 6) included "*Calculating Dose-volume and dilution of the drug"* functionalities in their systems. Another function mentioned for IV drugs was *"calculating the drug rates of infusion."*

Another functionality mentioned in most studies (*n* = 7) was *"using alarms and alerts in the system."* Studies in various pediatric and neonatal settings used this functionality to account for out-of-range doses. In addition, Siebert et al. [37] used this functionality as an alarm for validating the selection of look-alike or sound-alike medication.

### Risk of bias assessment data

#### The level of evidence results in the included papers

According to the level of evidence pyramid, half of the papers (*n* = 6) were CT. One was multicenter and crossover, located at the pyramid's highest point [37]. In this study, the randomization method was explicitly reported. An RCT crossover study was conducted in a medical center. Three studies at the third pyramid level were non-randomized CT crossovers. In the end, a non-randomized CT was conducted without any crossover.

The other category was observational studies (*n* = 3). A pretest–posttest study with a control group was conducted as a multicenter, at the highest level. Other papers in this category (*n* = 2) were a posttest with a control group and without a control group, located in the next levels, respectively. At the bottom of the pyramid were case studies (*n* = 3) that developed a system and introduced its components (Table 1).

#### Data collection methodologies and evaluated outcomes in the included papers

Half of the studies (*n* = 6) in simulated environments evaluated the rate of drug error and the time of drug preparation using the designed system compared to the traditional method. Four reported a reduced drug preparation time and error in medication administration.

A small number of studies (*n* = 3) evaluated the medication error rate in the real environment. Only one reported an improved MAE diagnosis. This study also calculated the time of patient exposure to harm due to drug error with and without the system [42]. Almost half of papers (*n* = 5) evaluated their system from the user’s perspective (Tables 1 and 3).

**Table 3:** Data collection methodology

**Data collection methodology**					
**Study design**	**Medication error calculation in the real environment**	**Medication error calculation in the simulated environment**	**Time and Motion Observed/Video Recording**	**Evaluation by** **Self-report/survey**	
**Platform**					
				**Usability** **Evaluation**	**User** **Perspective**
**RCT,** **Multicenter**		• Siebert et al. (2019) [37]	• Siebert et al. (2019) [37]		
		Mobile application			
**RCT,** **Crossover**		• Bury et al	• Bury et al. (2005) [38]		• Bury et al. (2005) [38]
		Web application			
**Nonrandomized** **CT, Crossover**		• Parush et al. (2020) [45]	• Parush et al. (2020) [45]	• Parush et al. (2020) [45]	
		Computer application	• Damhoff et al. (2014) [43]		
			• Ellis et al. (2012) [34]	• Ellis et al. (2012) [34]	
		• Damhoff et al. (2014) [43]			
		Web application			
		• Ellis et al. (2012) [34]			
		Computer application			
**Nonrandomized** **CT**		• Shannon et al. (2002) [35]	• Shannon et al. (2002) [35]		
		Web application			
**Pre-posttestcontrol,** **multicenter**	• Reynolds et al. (2019) [41]				• Reynolds et al (2019) [41]
	Mobile application				
**Posttest, control**	• Ateya et al. (2017) [44]				• Ateya et al. (2017) [44]
					Computer application
**Posttest**	• Ni et al. (2018) [42]				
	Web application, Mobile application, Computer application				
**Case study,** **development**					• Dodson et al. (2021) [36]
					Mobile application
					• Zahn et al. (2021) [39]
					Web application

## Discussion

The present systematic review aimed to identify the data elements and functionalities of existing MACDSSs in pediatrics and neonates. The identified data elements were mapped onto the FHIR standard. To sum up, the findings showed that the studies used a variety of data elements in system development. In total, 84 data elements were reported in these studies to map the FHIR standard. The functionalities reported in studies showed that 18 functionalities were implemented in their systems. The included studies showed that a very common functionality in these systems was *"Drug dosage calculation."* Besides, researchers designed their systems based on different platforms. However, most of them used mobile applications.

The analysis showed that the scope of most existing systems was limited to a specific category of drugs for pediatrics and neonates, and most of these systems were implemented standalone. Systems such as PedAMINES only contained resuscitation drugs [37]. LISA contained pediatric chemotherapy drugs [38]. Ateya et al. only calculated the insulin dose by developing an insulin calculator in a pediatric hospital [44].

The present findings showed that a few integrated MACDSSs were designed in real and unreal environments for pediatrics and neonates. The overall findings showed that most MACDSSs were standalone and used a comprehensive set of data elements and functionalities in their systems. A standalone design can reduce the use of the system [41]. Integrating these systems with other information technology infrastructures in hospitals can facilitate nurses’ acceptance of these systems. It can also reduce the MAE and facilitate the medication administration process in pediatrics and neonates.

The present findings revealed that the included papers did not use or report a standard format for identifying the data elements and functionalities for designing MACDSS in pediatrics and neonates. The lack of a standard format for designing such systems can prevent the entrance of the same data elements into the system. It further challenges reusing or sharing data with other systems [51]. Souza Pereira et al., in their systematic review, drew attention to the interoperability of CDSS design [15]. Our study presented a diverse list of data elements. Yet, their mapping onto the FHIR standard showed most of these data elements had been included in the existing standards. Therefore, developers need to use current standards as well as the present study that mapped the data elements onto the FIHR in the initial design of their systems.

Besides variation in data elements, the present study showed systems designed in medication administration for pediatrics and neonates did not use the same functionalities. Use of different functionalities in these systems can affect nurses' workflow, especially when they provide services in various institutions. In line with these findings, studies on CPOE or CDSS have also shown that these systems use a variety of functionalities [52, 53]. Furthermore, this systematic review assessed the level of evidence and outcomes in the included studies. These results showed that studies with a high level of evidence, for example, the one conducted by Siebert et.al. [37] used a comprehensive set of data elements and functionalities in their systems. Although observational studies were located at the second level, they were the only type that evaluated their systems in a real environment.

MACDSSs can be used to improve nurses’ and other users’ learning. Systems should have a set of functionalities to achieve this aim. One suggested functionality is *"Providing additional information about medicine to the user."* Miller et al. introduced this functionality for CDSSs in their review [54]. This functionality in information systems can be a helpful tool for educating nurses and nursing students, young physicians, and medical students, and furthering clinicians’ education.

The use of images and graphs in CDSSs can attract more user attention. Using images in a crowded and busy health environment can help better understand and interpret information. In their study, Miller et al. emphasized the importance of this issue in designing CDSSs [54]. Studies included in this systematic review also mentioned a functionality known as *"Providing a visual image and color text in the system".*

The present findings showed a significant gap in the existing systems using terminologies and standards such as RxNorm, HL7, and SNOMED CT. However, many studies recommended using standard terminologies in CDSS [55–60]. Standard terminologies can contribute to simplicity, consistency, and efficient interactions [54]. The present study revealed only three studies to report the use of standard terminologies in MACDSSs.

The present study is the SLR and studies with different methodologies were included. One strength is that to assess the quality of papers, the level of evidence method was used to report the quality of included studies.

An extended time was set for the search (i.e., 1995–2021). The majority of newer studies used mobile phones or tablet computers as the platform. Medication administration is usually done by a nurse at the point of care. Using portable platforms can improve the acceptance of systems by nurses and help reduce MAE.

The main scope of this study was to identify data elements and functionalities of MACDSSs in pediatrics and neonates. However, there were certain limitations in extracting data elements and functionalities in these systems. Primarily, the focus of the papers reviewed was specific drug categories; on the other hand, system components were reported less in manuscripts. For this reason, as far as possible, we examined the concepts and explanations provided in papers and the tables and figures to extract data elements and functionalities. We managed to identify a variety of data elements. To summarize and structure them, we used the FIHR standard to achieve a standard classification of findings.

The results retrieved a small number of studies in MACDSSs. However, we used a comprehensive search strategy with numerous keywords on multiple databases. Overall, this study managed to identify a list of numerous data elements and functionalities from the included studies.

MACDSSs developers in pediatrics and neonates can use the present findings for a better and more accurate design at the beginning of their system design process. A comprehensive set of data elements and functionalities helps them design a system tailored to their specific needs and objectives. With the right tools and approach, users can leverage the power of MACDSSs to make more informed decisions and achieve better outcomes. The authors recommend developers that used standard terminology such as RxNorm, HL7, and SNOMED CT before designing the MACDSSs for easy interoperability and sharing of data. General functionalities for CDSS can be used besides specific functionalities for MACDSS in pediatrics and neonates in the design process to reduce the potential rate of errors in medication administration and facilitate the drug preparation stage for nurses. Furthermore, they can design the new MACDSS using portable platforms to improve the acceptance of systems by nurses.

## Conclusion

The present systematic review provided a list of data elements and functionalities of MACDSSs systems in neonates and pediatrics. It identified the current platforms used in designing these systems. Identifying the data elements and functionalities used as a roadmap by developers can significantly improve the performance of MACDSSs.

Although many CDSSs have been developed for pediatrics and neonates in different medication processes (e.g., medication prescription), the present small retrieval of papers indicated that a small number of papers addressed the medication administration process in pediatrics and neonates. Studies that used such systems in real settings were scarce; thus, more research is required on using and evaluating MACDSSs in the real environment.

### Supplementary Information

**Additional file 1: S Table 1.** Data Elements for MACDSS.

**Additional file 2: S File 1.** PRISMA 2009 Checklist.

**Additional file 3: S File 2.** Search Strategy in CINAHL.

**Additional file 4: S File 3.** Functionalities Definition.

## Data Availability

The datasets used and/or analyzed during the current study are available from the corresponding author upon reasonable request.

## Abbreviations

FHIR: Fast Healthcare Interoperability Resources
MACDSS: Medication administration clinical decision support system
CDSS: Clinical decision support system
ADE: Adverse drug events
PRISMA: Preferred Reporting Items for Systematic Reviews and Meta-analyses
SLR: Systematic literature review
MeSH: Medical subject headings
EMTREE: Embase subject headings
HIT: Health information technology
RCT: Randomized control trials
CT: Clinical trials
