# Supplementary material for: Identifying the data elements and functionalities of clinical decision support systems to administer medication for neonates and pediatrics: a systematic literature review
Source: BMC Med Inform Decis Mak. 2023 Nov 16;23:263. doi: 10.1186/s12911-023-02355-5 (PMC10652533; doi:10.1186/s12911-023-02355-5)
Supplement: Supplementary file 1 — Additional file 1: S Table 1. Data Elements for MACDSS. [file 12911_2023_2355_MOESM1_ESM.docx]

| **Data Elements for MACDSS** |
| --- |
| **Category: Base** |
| \| Individuals \| \| --- \| \| \| Setting \| Resource name: Patient \| \| --- \| --- \| \| Data Element \| \| \| 1,2,3,4,5 \| Patient. Identifier \| \| 1,2,3,4,5 \| Patient. gender \| \| 1,2,3,4,5 \| Birth date (Age) \| \|  \| Individuals \| \| \| --- \| --- \| \| Setting \| Resource name: PractitionerRole \| \| Data Element \| \| \| 1,2,3,4 \| PractitionerRole \| \| 1,2,3,4 \| PractitionerRole.identifier \| \| 1,2,3,4 \| PractitionerRole. Practitioner \| \| 1,2,3,4 \| PractitionerRole.organization \| \| 1,2,3,4 \| PractitionerRole. Code \| \| 1,2,3,4 \| PractitionerRole. Healthcare service \|  \| Individuals \| \| --- \| \| \| Setting \| Resource name: Practitioner \| \| --- \| --- \| \| Data Element \| \| \| 1,2,3,4 \| Practitioner. Identifier \| \| 1,2,3,4 \| Practitioner.name \| \| 1,4 \| Practitioner. Password* \| \| 1,4 \| Practitioner. Organization* \| \|  \| **Entities#1** \| \| \| --- \| --- \| \| Setting \| Resource name: Organization \| \| Data Element \| \| \| 1 \| Type \|  \| **workflow** \| \| --- \| \| \| **Resource name:** [**Verification Result**](https://www.hl7.org/fhir/verificationresult.html) \| \| \| --- \| --- \| \| **Setting** \| **Data Element** \| \| 1,2,3,4,5 \| VerificationResult.need \| \| 1,2,3,4,5 \| VerificationResult.status \| \| 1,2,3,4,5 \| VerificationResult.status date \| \| 1,2,3,4,5 \| VerificationResult.validation type \| \| 1,2,3,4,5 \| VerificationResult.validation process \| \| 1,2,3,4,5 \| VerificationResult.failure actions \| \| 1,2,3,4,5 \| Verification Result. primarySource.who \| \| 1,2,3,4,5 \| VerificationResult.primarySource.validation Status \| \| |

**S Table 1: Data Elements for MACDSS**

| **Data Elements for MACDSS** |
| --- |
| **Category: Clinical** |
| \| **Medications** \| \| \| --- \| --- \| \| **Resource name: MedicationAdministration** \| \| \| **Setting** \| **Data Element** \| \| 1,2,3,4,5 \| MedicationAdministration \| \| 1,2,3,4,5 \| MedicationAdministration. Instantiates \| \| 1,2,3,4,5 \| MedicationAdministration.partOf \| \| 1,2,3,4 \| MedicationAdministration. Status \| \| 1,2,3,4 \| MedicationAdministration. Status reason \| \| 1,2,3,4,5 \| MedicationAdministration.medication \| \| 1,2,3,4,5 \| MedicationAdministration.category \| \| 1,2,3,4,5 \| MedicationAdministration. Subject \| \| 1,2,3,4,5 \| MedicationAdministration.supporting information \| \| 1,2,4 \| MedicationAdministration.effective \| \| 1,2,3,4,5 \| MedicationAdministration.performer \| \| 1,2,3,4 \| MedicationAdministration.performer.function \| \| 1,2,3,4,5 \| MedicationAdministration.performer.actor \| \| 1,2,3,5 \| MedicationAdministration.dosage \| \| 1,2,3,4 \| MedicationAdministration.event history \| \|  \| \| \|  \| **Resource name: MedicationKnowledge** \| \|  \| **Data Element** \| \| 1,2,3,4,5 \| MedicationKnowledge \| \| 1,5 \| MedicationKnowledge.MedicineClassification \| \| 1,4,5 \| MedicationKnowledge. Administration guidelines \| \| 1,4,5 \| MedicationKnowledge.administration guidelines. Dosage \| \| 1,2,3,4,5 \| MedicationKnowledge.administration guidelines. Patient characteristics \| \| 1,4,5 \| MedicationKnowledge.code \| \| 1,2,3,4,5 \| MedicationKnowledge.relatedMedicationKnowledge \| \| 1,2,3,4, \| MedicationKnowledge.preparationInstruction \| \| 1,2,3,4 \| MedicationKnowledge.intended route \| \| 1,2,3,5 \| MedicationKnowledge.drug characteristic \| \| 1,2,3,4,5 \| MedicationKnowledge.contraindication \| \| 1,2,3,4,5 \| MedicationKnowledge.regulatory \|  \| **Medications** \| \| \| --- \| --- \| \|  \| \| \|  \| \| **Resource name: Medication** \| \| \| --- \| --- \| \| **Setting** \| **Data Element** \| \| 1,2,3,4,5 \| Medication \| \| 1,2,3,4,5 \| Medication. identifier \| \| 1 \| Medication. code \| \| 1,2,3,5 \| Medication. Status \| \| 1,2,3,5 \| Medication. Form \| \| 1,2,3,4,5 \| Medication. amount \| \| 1 \| Medication. Barcode* \| \| 1,2,3,5 \| Medication References* \| \| 1,2,3 \| Dissolvent Name* \| \| 1,2,3 \| Dissolvent Dosage Range* \| \| 1,2,3 \| Dissolvent Dosage* \| \| 1,2,3 \| Dissolvent Dosage Unit* \| \| 1,2,3,5 \| Side Effect* \| \|  \| **Modifier Extension: Dosage** \| \| 1,2,3,4,5 \| Dosage. sequence \| \| 1,2,3,4,5 \| Dosage. text \| \| 1 \| Dosage. timing \| \| 1,2,3 \| Dosage. route \| \| 1,2,3 \| Dosage. method \| \| 1,2,3,4,5 \| Dosage.dose And Rate \| \| 1,2,3 \| Dosage. Dose And Rate. Rate \| \| 1,2,3,4,5 \| Dosage.maxDosePerPeriod \| \| 1,2,3,4,5 \| Dosage.maxDosePerAdministration \| \|  \| **Care provision** \| \| \| --- \| --- \| \| **Resource name: Care Team** \| \| \| **Setting** \| **Data Element** \| \| 1,2,3,4,5­ \| Name \|  \| **Diagnostics** \| \| --- \| \| \| **Resource name: Observation** \| \| \| \| \| --- \| --- \| --- \| --- \| \| **Setting** \| **Data Element** \| \| \| \| 1,2,3,4,5 \| Weight \| \| Vital  Signs \| \| 2,4 \| Height \| \| \| 2,4 \| Body Surface Area \| \| \|  \| \| \| \| \| **Resource name: DiagnosticReport** \| \| \| \| \| **Setting** \| \| **Data Element** \| \| \| 1,2,3,4,5 \| \| DiagnosticReport.result \| \| \| \| **Resource name: Media** \| \| \| --- \| --- \| \| **Setting** \| **Data Element** \| \| 1,2,3,5 \| Media \| \| \| \| \| \|  \| **Request & Response** \| \| \| --- \| --- \| \| **Resource name: Communication** \| \| \| **Setting** \| **Data Element** \| \| 1,2,3,4,5 \| Communication. category \| \| 1,2,3,4 \| Communication. medium \| \| 1,2,3,4 \| Communication. topic \| \| 1,2,3,4 \| Communication. sent \| \| 1,2,3,4 \| Communication. received \| \| 1,2,3,4 \| Communication. recipient \| \| 1,2,3,4 \| Communication. sender \| \| 1,2,3,4 \| Communication. payload \| \| 1,2,3,4 \| Communication. note \|   *The data elements are related to the researcher extension.  Pediatric emergency:1  NICU:2  PICU:3  Pediatric chemotherapy:4  General pediatric:5 |
