# Supplementary material for: Identifying the data elements and functionalities of clinical decision support systems to administer medication for neonates and pediatrics: a systematic literature review
Source: BMC Med Inform Decis Mak. 2023 Nov 16;23:263. doi: 10.1186/s12911-023-02355-5 (PMC10652533; doi:10.1186/s12911-023-02355-5)
Supplement: Supplementary file 3 — Additional file 3: S File 2. Search Strategy in CINAHL. [file 12911_2023_2355_MOESM3_ESM.docx]

**S File 2: Search Strategy in CINAHL**

**Category1**:

(TX “Clinical Decision Support System*” OR TX “Clinical Decision Support*” OR TX “Clinical Decision Support tool*” OR TX “Handheld decision support” OR TX “Drug Information System*” OR TX (“Alert System*” AND Medication) OR TX “Medication Alert System*” OR TX (System AND “Medication Alert”) OR TX (“Alert Systems” AND Medication) OR TX “Mobile Application*” OR TX “Mobile Apps” OR TX “Mobile App” OR TX “reminder system*” OR TX “dosage calculat*” OR TX “web based” OR TX “web-based” OR TX “automated system”)

AND

**Category2**:

(TX “Drug Dosage Calculations” OR TX “Drug calculation“ OR TX “Drug Administration Routes” OR TX “Drug administration” OR TX “Drug Route” OR TX “Drug delivery” OR TX “Drug handling” OR TX “Drug preparation” OR TX “Medication calculation” OR TX “Medication administration” OR TX “Medication handling” OR TX “Medication delivery” OR TX “Medication preparation” OR TX “Medicine preparation” OR TX “Medicine administration” OR TX “Medicine delivery” OR TX “Medicine handling” OR TX “Medication Route” OR TX “Dose calculation*” OR TX “Doses calculation*” OR TX “Medication dose”) AND (PY 1995-2021)
