# Supplementary material for: Identifying the data elements and functionalities of clinical decision support systems to administer medication for neonates and pediatrics: a systematic literature review
Source: BMC Med Inform Decis Mak. 2023 Nov 16;23:263. doi: 10.1186/s12911-023-02355-5 (PMC10652533; doi:10.1186/s12911-023-02355-5)
Supplement: Supplementary file 4 — Additional file 4: S File 3. Functionalities Definition. [file 12911_2023_2355_MOESM4_ESM.docx]

**S File 3: Functionalities Definition**

1. **Drug dosage calculation**

Math calculations are done for preparing appropriate doses of medicines, taking into account conversions of weight and other measures.

1. **Calculating Dose volume**

It calculates and administers the dose of medicine based on its volume, e.g., milliliters. **Calculating dilution of drug**: Math calculations that are done for diluting medicine. Dilution decreases the concentration of a medicine, usually simply by mixing with more solvent, like adding more water to the medicine.

1. **Calculating drug rates of infusion**

Calculating infusion rates is simply dividing the volume of fluid being administered by the time over which it is given.

1. **Keeping history of information in the system**

Keeping a history of information is the act of maintaining the history of patient care, generally by creating and storing consistent, formal records. It consists of detailing all aspects of patient care.

1. **Use alarms and alerts in the System & Type of alert**

An alert is any signal that draws attention to something. An alarm is a short warning, drawing instant attention to some danger.

1. **Displaying the status of stages of medication use process**

Five phases of the medication use process (prescribing, transcribing, dispensing, administering and monitoring) which display in the system.

1. **Having the capability to share information with caregivers**

The capability to electronically share information between caregivers and institutions. e.g., Sharing the list of edited formulations of drugs between caregivers and institutions is possible through upload and download options via identity codes created by the list owner. (Siebert et al. (2019))

1. **Having the capability to edit drug information in the system**

The capability to allow users to edit drug information in the system. e.g., the app contains a drug editor that allows users to create exhaustive lists of drugs which they use in their settings and to edit them according to local formulations and habits. (Siebert et al. (2019))

1. **Providing a visual image and color text in the system**

It means displaying visual images and color text in the system. e.g., iDoseCheck (Ellis et al. (2013)) provides a visual image of the number of vials of morphine to use and a visual image of the correct amount of drug in the appropriate syringe.

1. **Providing additional information about medicine to the user**

The capability to provide additional information about medicine to the user. e.g., information about adverse drug events.

1. **Having the capability to clear information in the system**

This icon is about the system's capability to clean or delete information in the system.

1. **Having the capability to search in the system**

A search box is a graphical element present in many applications and websites. It acts as the field for a query input or search term from the user to search and retrieve related information from the database.

1. **Having the capability to log in/out in the system**

[You are connect](https://dictionary.cambridge.org/dictionary/english/connect)ing to a [system](https://dictionary.cambridge.org/dictionary/english/system) by putting in a [particular](https://dictionary.cambridge.org/dictionary/english/particular) set of [letters](https://dictionary.cambridge.org/dictionary/english/capital) or [numbers](https://dictionary.cambridge.org/dictionary/english/number) [and](https://dictionary.cambridge.org/dictionary/english/system) by typing your name so that you can [start](https://dictionary.cambridge.org/dictionary/english/start) [working](https://dictionary.cambridge.org/dictionary/english/working).

1. **Having the capability to lookup by barcode in the system**

Search for a drug by barcode number using the search box.

1. **Having the capability to modify dose in the system**

The modifications made to a medication dose, e.g., decrease the dose, increase the amount. The PedAMINES (Siebert et al. (2019)) has developed this functionality with ±icon.

1. **Displaying drug administration route:**

The route of administration is the way the dosage form is given. Common routes of administration include IV/IO, IM, IN.

1. **Displaying dose form/ dosage regimen:**

The dosage form is the physical form of a dose of a drug. Typical dosage forms include tablets, capsules, etc. The strength is the amount of medicine in the dosage form or a unit of the dosage form (e.g., 0.3 mcg/kg/min Epinephrine). The dosage regimen is the frequency at which the drug doses are given. Examples include 2.5 mL twice a day, one tablet three times a day, one injection every four weeks.

1. **Displaying medication preparation steps**

Describes the step/task/action that is within a medication preparation.
